# Driver, Collision and Meteorological Characteristics of Motor Vehicle Collisions among Road Trauma Survivors

**DOI:** 10.3390/ijerph182111380

**Published:** 2021-10-29

**Authors:** Melita J. Giummarra, Rongbin Xu, Yuming Guo, Joanna F. Dipnall, Jennie Ponsford, Peter A. Cameron, Shanthi Ameratunga, Belinda J. Gabbe

**Affiliations:** 1 Department of Epidemiology and Preventive Medicine, School of Public Health and Preventive Medicine, Monash University, Melbourne, VIC 3004, Australia; rongbin.xu@monash.edu (R.X.); yuming.guo@monash.edu (Y.G.); jo.dipnall@monash.edu (J.F.D.); peter.cameron@monash.edu (P.A.C.); belinda.gabbe@monash.edu (B.J.G.); 2 Caulfield Pain Management and Research Centre, Caulfield Hospital, Caulfield, VIC 3162, Australia; 3 Mental and Physical Health and Clinical Translation (IMPACT), School of Medicine, Deakin University, Geelong, VIC 3220, Australia; 4 School of Psychological Sciences, Monash University, Clayton, VIC 3800, Australia; jennie.ponsford@monash.edu; 5 Monash-Epworth Rehabilitation Research Centre, Epworth Hospital, Richmond, VIC 3121, Australia; 6 School of Population Health, University of Auckland, Auckland 1010, New Zealand; s.ameratunga@auckland.ac.nz; 7 Population Health Directorate, Counties Manukau District Health Board, South Auckland 2104, New Zealand; 8 Health Data Research UK, Swansea University Medical School, Singleton Park, Swansea University, Swansea SA2 8PP, UK

**Keywords:** motor vehicle, prevention, traffic, trauma

## Abstract

Road trauma remains a significant public health problem. We aimed to identify sub-groups of motor vehicle collisions in Victoria, Australia, and the association between collision characteristics and outcomes up to 24 months post-injury. Data were extracted from the Victorian State Trauma Registry for injured drivers aged ≥16 years, from 2010 to 2016, with a compensation claim who survived ≥12 months post-injury. People with intentional or severe head injury were excluded, resulting in 2735 cases. Latent class analysis was used to identify collision classes for driver fault and blood alcohol concentration (BAC), day and time of collision, weather conditions, single vs. multi-vehicle and regional vs. metropolitan injury location. Five classes were identified: (1) daytime multi-vehicle collisions, no other at fault; (2) daytime single-vehicle predominantly weekday collisions; (3) evening single-vehicle collisions, no other at fault, 36% with BAC ≥ 0.05; (4) sunrise or sunset weekday collisions; and (5) dusk and evening multi-vehicle in metropolitan areas with BAC < 0.05. Mixed linear and logistic regression analyses examined associations between collision class and return to work, health (EQ-5D-3L summary score) and independent function Glasgow Outcome Scale - Extended at 6, 12 and 24 months. After adjusting for demographic, health and injury characteristics, collision class was not associated with outcomes. Rather, risk of poor outcomes was associated with age, sex and socioeconomic disadvantage, education, pre-injury health and injury severity. People at risk of poor recovery may be identified from factors available during the hospital admission and may benefit from clinical assessment and targeted referrals and treatments.

## 1. Introduction

In 2000, the state of Victoria, Australia, implemented the Regionalised Victorian State Trauma System, which led to significant improvements in survival after serious injury [1]. Further advances in trauma triage, healthcare and treatment since then have continued to lead to improved functional outcomes for survivors of serious injury [2]. However, road trauma remains a significant public health problem in Victoria, as it does globally [3], where transport-related mechanisms account for 39% of all serious injury [4]. The economic burden of hospitalised road trauma is estimated atAUD 29.7 billion each year in Australia [5], with significant impacts on healthcare use [6], compensation scheme costs [7] and reductions in work participation [8] and quality of life [9,10]. It is therefore important that we seek to better understand the complex circumstances of collisions resulting in serious injury from road trauma, and their associations with injury outcomes, so that we can reduce the impact of road trauma.

Most of the literature to date has characterised road trauma using relatively simple methods examining associations between key individual, behavioural and environmental risk characteristics and rates of motor vehicle collisions and mortality. Few studies have examined road trauma characteristics using person-oriented modelling, which enable us to identify patterns across multiple characteristics for individual cases within a dataset [11]. An exception was a study in Sweden, published more than ten years ago, that used cluster analysis to examine collision characteristics in young drivers [12]. They found five main types of collisions: (1) single-vehicle collisions in sparsely populated areas with speed limits >70 km/h; (2) front-on collisions in daylight and on icy surfaces, particularly in older vehicles travelling > 90 km/h; (3) crashes at dawn or dusk, typically with no other vehicle counterpart in sparsely populated areas; (4) turning vehicles, typically in later model cars during daylight with speed limits <50 km/h; and (5) crashes in urban areas with speed limits below 50 km/h, including turning or rear-end collisions and impacts with objects or pedestrians, cyclists or animals. Given that there is enormous variation in road infrastructure, legislation, geography and other localised factors (e.g., native animal behaviours), it is difficult to extrapolate these findings to other jurisdictions. Therefore, the primary aims of the present study were to (a) identify and characterise sub-groups of collisions resulting in hospitalisation for serious injury using latent class analysis in Victoria, Australia, and (b) determine whether understanding collision circumstances contributes to the prediction of patient outcomes. As we focused on the association between collision circumstances and outcomes, we only included drivers who survived the first 12 months after injury.

## 2. Materials and Methods

### 2.1. Participants

People aged ≥16 years with serious transport-related injury between 1 July 2010 and 30 June 2016 were included from the Victorian State Trauma Registry (VSTR). Eligibility criteria included being injured as a motor vehicle driver and having a compensation claim with Victoria’s no fault transport injury compensation system, the Transport Accident Commission (TAC). People were excluded if their injury was deemed intentional by the hospital coders, if they sustained a severe head injury (head Abbreviated Injury Scale severity score > 2 and GCS of 3–8) or if they died ≤12 months after injury.

### 2.2. Setting, Data Sources and Data Linkage

The VSTR is a population-based registry that collects information on all patients who meet major trauma criteria and are admitted to one of 138 trauma receiving health services in the Victorian State Trauma System in Australia [13]. Major trauma is defined as: (1) death after injury; (2) admission to intensive care for >24 h and requiring mechanical ventilation for at least part of their stay; (3) Injury Severity Score (ISS) > 12; or (4) surgery within 48 h for intracranial, intrathoracic or intraabdominal injury, or for fixation of pelvic or spinal fractures. The VSTR collects demographic, prehospital and injury-related data from the hospital records and hospital coders. Additional demographics and outcomes are collected in structured telephone interviews with patients or proxy respondents at 6, 12 and 24 months post-injury. Post-discharge death dates are identified through linkage with the Victorian Registry of Births, Deaths and Marriages.

The TAC is a Victorian state government-owned organisation that supports people who are injured in incidents involving motorised vehicles, trains or trams in Victoria, Australia. All claimants are entitled to statutory benefits, regardless of fault, for a range of supports including healthcare and income replacement. Benefits may also include lump sum payments if the claimant sustained impairment greater than 10%, as determined in an Independent Medical Examination (IME) in accordance with the American Medical Association Guides for the provision of Impairment Assessment [14]. Claimants with contributory negligence, such as drivers with a blood alcohol concentration (BAC) of ≥0.05 mmol ethanol per litre, have restricted benefit entitlements. Claimants are also entitled to common law benefits if another party was partially or completely at fault, and the claimant was seriously injured. Claimant information for participants, as well as other claimants injured in the same injury event, were obtained from the TAC via the routine data linkage between the VSTR and TAC.

Weather characteristics during each injury event were identified through linkage with half-hourly weather recordings from the Australian Bureau of Meteorology. Sunrise and sunset times were collected from Geosciences Australia recordings to determine the sunshine-related visibility conditions at the time of day in which each injury event occurred.

### 2.3. Demographic Characteristics

Participant demographic characteristics were obtained from the VSTR, including age at injury, sex, preferred language, education level, work status and occupation pre-injury, and residential postcode at the time of injury. Age was categorised into ranges given that studies typically find a non-linear association between age and injury outcomes [7]. Education level was classified according to the Australian Standard Classification of Education [15]. Occupation skill level was classified into four levels in accordance with the Australian Standard Classification of Occupations [16]: managers, professionals and associate professionals; tradespersons and advanced clerical workers; intermediate sales, clerical, service, production and transport workers; and elementary sales, clerical and service workers and labourers.

Residential postcodes were used to link the Index of Relative Socioeconomic Advantage and Disadvantage (IRSAD) deciles [17] and Accessibility/Remoteness Index of Australia (ARIA) classifications [18] from the 2016 census. The IRSAD is based on national census data on the typical family structure, employment and education level within each postcode region. The Victorian ranked IRSAD deciles were summarised into quintiles ranging from one (most disadvantaged) to five (least disadvantaged). The ARIA classifies regions in Australia into five levels of remoteness (major cities, inner regional, outer regional, remote, very remote), which were summarised as major cities versus regional and remote areas due to the small number of remote regions in Victoria.

### 2.4. Pre-Injury Health Characteristics

International Classification of Diseases (10) Australian Modification (ICD-10-AM) diagnosis codes were used to generate the Charlson Comorbidity Index (CCI) [19], and to identify comorbid substance use or mental health conditions in accordance with published criteria [20]. The CCI includes comorbid conditions that increase mortality risk, and higher weightings indicate higher mortality risk. Disability level in the week before injury was collected in the follow-up interviews, which has been found to have satisfactory consistency with ratings at the time of hospital admission [21], using a five-level rating scale ranging from no disability to severe disability.

### 2.5. Injury Characteristics

Injury characteristics included the ISS [22], injured body regions based on the maximum AIS 2005 (2008 revision) severity scores, length of hospital stay and discharge destination. Using the AIS body region severity scores, injuries were classified into groups based on the most common patterns of injured body regions, with one additional group for patients with burns or multiple injuries but without serious neurotrauma, as per previous studies [23]. A variable indicated whether drivers had a BAC ≥ 0.05 mmol ethanol per litre at hospital admission.

### 2.6. Collision Characteristics

Data from the TAC were used to identify collision characteristics that represented the complexity and characteristics of the collision, including the number of vehicles and claimants in the collision, and whether others sustained serious injury. Other claimants were identified via the TAC “accident key”. Serious injury in other claimants was defined as (a) death from injury; (b) injury involving paraplegia, quadriplegia or moderate to severe brain injury; (c) injury resulting in an impairment level ≥30% from an IME report to the TAC; or (d) being classified as having a catastrophic injury or “other severe” injury by the TAC.

Fault attribution of the study participant was collected by the TAC in the claim lodgement process, and from police. We used the claimant’s attribution, which was supplemented with the police attribution of fault if the claimant did not know who was at fault, and identified whether claimants denied or claimed fault of another party if their attribution differed from the police assessment. For the latent class analyses, people who recorded unknown fault were allocated to the group that did not attribute fault to another. This does not mean that the person accepted personal responsibility, but that they did not believe that another person was at fault.

### 2.7. Weather and Time of Day

The time of day at which the injury event occurred was classified in relation to sunrise and sunset times obtained from Geosciences Australia, based on the latitude and longitude for the centroid of the postcode where the injury event occurred. The precise locations of injury events were not available to protect participant anonymity. Time of day was categorised as: ≤1 h before sunrise; 0 to 1 h after sunrise; daylight (>1 h after sunrise and >1 h before sunset); ≤1 h before sunset; 0–1 h after sunset; and evening/early morning when it was dark (>1 h after sunset and >1 h before sunrise). These criteria were consistent with previous studies examining injury in relation to daylight hours, including the separation of the hours before and after sunrise and sunset, where lighting conditions and the position of the sun on the horizon can have an increased influence on safety [24,25].

Weather conditions at the time and location of the collision based on injury postcode included the presence of any precipitation, the level of wind gusts (km/h) and visibility (km). Inclement conditions were defined as precipitation > 0.10 mm/h, wind gust ≥ 3.0 km/h or visibility < 1 km, consistent with previous studies [26]. For each case, the weather data were generated using the weather observations of 260 weather stations in and around Victoria for every 30 min from 2010 to 2016, and the two half-hourly observations that were closest to the time and postcode where the collision occurred (e.g., for 9.10 am, we chose observations at 9.00 am and 9.30 am). For each station, the weather condition at time of the collision was then estimated by linear interpolation of the two closest half-hourly observations assuming a linear trend between the two time points. For the 16 cases for which no time of injury was recorded, the station-specific daily average observations during the day of injury were used (i.e., the average of all half-hourly observations). The weather conditions (precipitation, wind gust and temperature) in the postal area where the injury occurred were estimated by inverse distance weighting spatial interpolation based on all station-specific observations using previously developed algorithms [27,28]. This final step accounted for the recordings from all weather stations to estimate the most likely conditions in the geographic area where the collision occurred. The postal areas were defined according to the Australian Statistical Geography Standard 2016. For visibility data, station-specific observations set all records higher than 10 km as 10 km. Therefore, it was not appropriate to perform spatial interpolation for visibility based on these records. Instead we used the observation that was closest to the centroid of the postal area at the time of the collision to represent the visibility conditions.

### 2.8. Outcome Variables

Outcomes from follow-up interviews at 6, 12 and 24 months post-injury included return to work or study, health status using the EQ-5D-3L summary score [29] and independent function on the Glasgow Outcome Scale-Extended (GOS-E) [30]. Return to work or study was recorded for people who were working or studying before injury. The EQ-5D-3L utility score was calculated using an adaptation of the original syntax by Viney et al. [31] with Australian Tariffs [32]. A score of 1.00 indicates perfect health, 0.00 is equivalent to death and scores < 0.00 indicate a health state worse than death. The GOS-E scores were dichotomised as independent living (ratings of 5 to 8) or severe disability or death (ratings of 1 to 4).

### 2.9. Data Analysis

**Figure 1:**
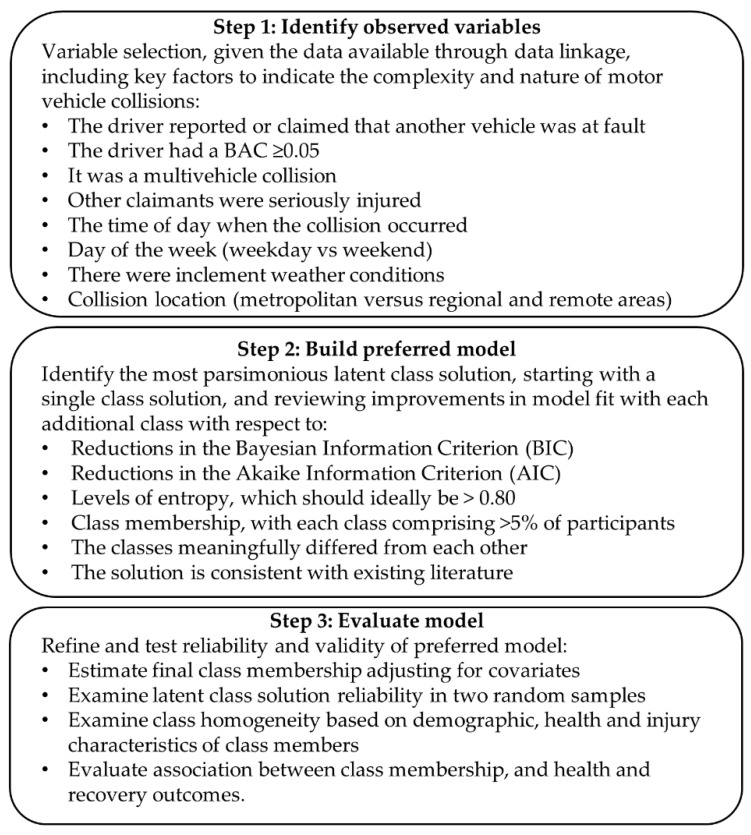
Flow chart of the latent class analysis methods.

All analyses were completed using Stata (Version 15.0, College Station, TX, USA: Stata Corporation). The key steps for the latent class analysis (LCA) are summarised in Figure 1. Variable selection was limited by the nature of the data available from the registries, and the linked administrative and geospatial meteorological data. Characteristics known to play a role in collision risk, such as road speed, vehicle speed, vehicle characteristics or other driver behaviours, were not available. A related study that analysed the text description of the injury event for the included cases showed that very people few reported such characteristics in their collision description in their TAC claim [33].

The LCA plugin for Stata, version 1.2.1 developed by Lanza et al [34], Pennsylvania State University, Pennsylvania, USA, was used to identify the probable number of collision classes, and to estimate class membership. LCA is a finite mixture modelling approach used to identify homogeneous groups, or classes, within a heterogeneous sample or population. It uses maximum likelihood estimation to generate a probabilistic model that identifies the most likely latent classes to describe the data. Key characteristics of each class are typically defined by the indicator variables that have very high or low posterior probabilities compared with the other classes. The LCA approach accommodates missing data through estimations of the expected characteristic given the observed items for that individual; however, cases with data missing for covariates are omitted. When evaluating model fit, we favoured model fit appraisals on flattening of the reduction in Bayesian Information Criterion (BIC) as it is considered to be more reliable than the Akaike Information Criterion (AIC) and entropy [35,36]. If a simplified version of the model improved fit, the process started again at Step 1. The sample was randomly split into two groups to test the reliability of the classes using the favoured LCA model parameters [37], after first confirming that both groups did not differ on key demographics (age and sex) or collision characteristics (i.e., the variables included in the LCAs). Homogeneity of the observed demographic, health and injury-related characteristics of class members was examined using chi square tests [36,38].

The associations between latent class membership and outcomes were examined using mixed logistic and linear analyses for binary (return to work; GOS-E outcomes) and continuous outcomes (EQ-5D summary score), respectively. Robust standard errors estimated 95% confidence intervals. Analyses modelled time (6 months, 12 months and 24 months), and a random intercept for participant identity, and included demographic, health and injury-related covariates. Multicollinearity was examined using the variance inflation factor (VIF), and no violations were found (i.e., all VIF values were <4.00, as recommended by Fox [39]). Multiple imputation using chained equations was used to estimate missing data for covariates, imputing and combining 20 datasets using the other covariates included in each model [40,41].

## 3. Results

### 3.1. Cohort Overview

**Figure 2:**
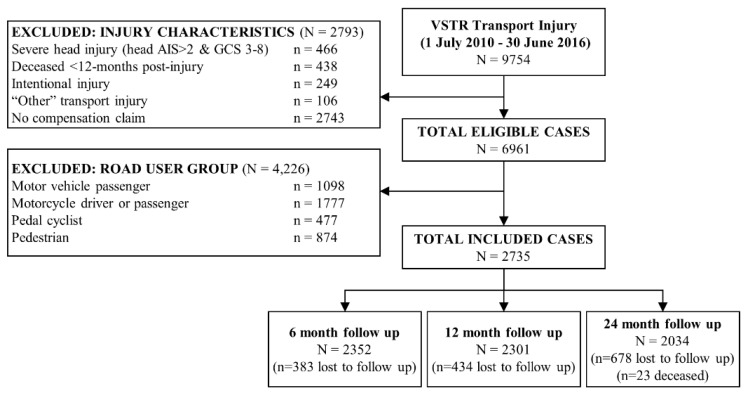
STROBE participant inclusion chart.

A total of 9754 major trauma admissions following road traffic injuries were registered to the VSTR from 1 July 2010 to 30 June 2016. Of these admissions, 7019 were excluded from this study (Figure 2). Of the 2735 motor vehicle drivers with a compensation claim who met the inclusion criteria, 2467 (90.2%) had data for one or more collision characteristic for inclusion in the latent class analyses, 2286 (92.7%) of whom were followed up at least once in the first two years post-injury. A higher proportion of cases who were lost to follow-up were younger, did not speak English as their preferred language, lived in neighbourhoods with greater disadvantage, had not completed secondary school, had a BAC ≥ 0.05 during the collision or a pre-injury substance use condition and were injured in collisions where no other was at fault (Table 1). BAC tests were not done for 878 cases, and results were not available for 367 cases.

The prevalence of injuries occurring on each day of the week was relatively consistent, ranging from 352 (12.9%) collisions occurring on Wednesdays to 431 (15.8%) collisions occurring on Fridays. Most collisions occurred without inclement weather, but 987 (36.1%) occurred during precipitation, 498 (18.2%) were in gusty conditions and 35 (1.3%) occurred when there was impaired driving visibility. The average temperature at the time of collision was 13.9 °C (SD = 5.8 °C), and 95% of collisions occurred at temperatures of <25.0 °C.

### 3.2. Latent Classes

The model with the best fit comprised five classes (Table S1), and adjusting for covariates of age and sex reduced log-likelihood indicating that the adjusted model improved model fit further. The key characteristics of each collision are detailed in Table 2, and the comparison of characteristics of people in each class is reported in Table S2.

Collision class 1 (*n* = 663, 30.2%) comprised daytime (100%) multi-vehicle (100%) collisions in which the majority of cases did not attribute fault to another vehicle (79%), 74% of which occurred on a weekday, and 98% of drivers had a BAC < 0.05. People in class 1 were older than all other classes (*m* = 59.75, SD = 19.63), were predominantly female (55.8%), had not completed high school or had an advanced diploma only (45.2% and 26.7%, respectively). Moreover, just over half of the people in class 1 were not working (56.1%). Compared with the other classes, a larger proportion of people in class 1 had a pre-injury disability (22.9%).

Collision class 2 (*n* = 600, 25.1%) comprised daytime (100%) single-vehicle (97%) collisions, 61% of which occurred in regional/remote areas, a quarter occurred in inclement weather, and 75.0% occurred on a weekday. People in class 2 were predominantly middle-aged (*m* = 49.18, SD = 20.73), male (64.7%) and either not working (37.1%) or working in lower elementary-trade skill level jobs (39.2%). Class 2 was the only class with more than half of its members living in regional or remote areas (52.5%).

Collision class 3 (*n* = 711, 25.9%) comprised evening (100%) single-vehicle (60%) collisions, 60% of which occurred on a weekday, 36% had a driver with a BAC ≥ 0.05, 33% involved inclement weather and 37% occurred in regional/remote areas. Most class 3 collisions had no other at fault (91%), and no other claimant who was seriously injured (86%). Class 3 members were younger than the other classes (*m* = 34.35, SD = 14.92) and were predominantly male (76.7%), lived in metropolitan areas (67.1%) and had lower education levels, but this class had the largest proportion of people with a CCI condition (29.8%), mental health condition (12.0%) or substance use (22.1%) condition pre-injury.

Collision class 4 (*n* = 365, 14.4%) appeared to comprise commuter collisions occurring at sunrise (19% before and 27% after sunset) or sunset (29% before and 25% after sunset), of which 75% occurred on a weekday, and 52% involved two or more vehicles. Only 28% of collisions occurred in inclement weather, 14% involved serious injury to another, and 16% of drivers had a BAC ≥0.05. Class 4 members were middle-aged (*m* = 42.12, SD = 18.86) and predominantly male (67.1%), and just over half lived in metropolitan areas (54.3%).

Collision class 5 (*n* = 128, 4.4%) comprised late day (before/after sunset) and evening multi-vehicle (100%) collisions in metropolitan areas (92%) where another was at fault (100%), with 30% occurring in inclement weather, 38% involving serious injury to one or more other claimant, and 77% occurring on a weekday. Class 5 members all had a BAC < 0.05. People in class 5 were middle-aged (*m* = 45.22, SD = 15.94), predominantly male (63.3%) and spoke English (99.0%). Compared with the other classes, the class 5 members comprised the largest proportion of people with an advanced diploma (43.5%) or working in trade-level occupations (24.2%), with no pre-injury disability (91.3%).

There were no significant differences between classes for preferred language, pre-injury substance use conditions or injury severity. Moreover, the temperature at the time and location of collisions did not differ meaningfully between classes, ranging from an average of 13.1 °C for collision class 5 to 14.6 °C for collision class 3, *p* = 0.01.

### 3.3. Class Solution Reliability

Two random samples comprising 1329 and 1406 cases showed consistent characteristics in each sub-sample with no covariates (Table S3). When adjusting for age and sex, classes one to three were very similar, but classes four and five had small changes to the proportion of cases injured in regional areas and at different times of the day (Table S4). Overall, the five-class solution appeared to be reliable, with some variability when accounting for driver age and sex.

### 3.4. Association between Collision Class and Outcomes

The mixed linear and logistic regression analyses showed that health, function and return to work all significantly improved over time, with six-fold higher odds of having returned to work, two-fold higher odds of returning to independent function and an average of 0.01 points higher health status on the EQ-5D-3L at 24 months post-injury compared with 6 months post-injury (Table 3). Compared with collision class 1 (predominantly multivehicle collisions with no other at fault), people in collision classes 2 and 4 who were predominantly injured in collisions that occurred in a single vehicle collision or during commuter hours had better health status outcomes (Table S5) and 2.8 and 2.4-fold higher odds of returning to work (Table S6) in unadjusted analyses, respectively. People in collision class 5 who were predominantly injured in metropolitan collisions in which another was at fault and in conditions with heightened collision risks (i.e., inclement weather and drivers with BAC ≥ 0.05) had 57% lower odds of reporting independent function in the follow-up interviews than people in collision class 1 in the unadjusted analyses (Table S7). Following adjustment for demographic, health and injury-related characteristics, however, the collision classes did not differ in health, functional or return to work outcomes (Table 3). When the analyses were adjusted for collision class membership, and all other available characteristics, better recovery outcomes were associated with younger age, male sex, higher socioeconomic position and education level, better pre-injury health (i.e., no CCI comorbid conditions or pre-injury disability), working in occupations with higher skill level and lower injury severity.

## 4. Discussion

This study provides novel insight into the most prevalent characteristics of collisions in a population-level cohort of drivers who were seriously injured and admitted to hospital in Victoria, Australia, over a six-year period. We identified five classes, summarised below, and demonstrate that rich insights into circumstances resulting in injury can be gained when examining individual-level patterns across datasets that include a range of linked data sources. Work, health and functional outcomes were not associated with collision class membership when accounting for their demographic, health and injury-related characteristics. The demographic, health and injury characteristics found to be associated with outcomes replicated the associations already known from the existing literature on the predictors of injury outcomes. Therefore, while applying classification person-level modelling offers new insights into the complex circumstances resulting in serious injury for motor vehicle drivers, those collision characteristics did not improve our understanding of longer-term outcomes in the present study. We note, however, that other collision-related characteristics that were not available in the present study would most likely have an impact on longer-term outcomes, particularly vehicle type, age and speed [12,42,43,44], which we discuss further in the study limitations below. Altogether, the study findings highlight that patient screening, assessment and treatment to support long-term recovery after motor vehicle collision should primarily focus on patient characteristics rather than the circumstances in which they were injured.

The majority of people belonged to the class in which collisions occurred in the evening with no other vehicle, which predominantly involved younger male drivers with a relatively high prevalence of substance use and other comorbid conditions (29% of all collisions, collision class 3). Daytime multi-vehicle collisions were the next most prevalent type of collision, and predominantly involved older female drivers and no other driver was at fault (27% of all collisions, collision class 1), and single-vehicle collisions predominantly in regional areas on weekdays (24% of all collisions, collision class 2). Collisions that occurred around sunrise or sunset on weekdays were less common and predominantly involved middle-aged male drivers (15% of collisions, collision class 4). Finally, the least prevalent collisions were those that occurred at the fault of another driver and predominantly occurred in the late afternoon or evening in metropolitan areas during inclement weather, and resulted in serious injury to other claimants compared with the other collision classes, with injured drivers having a low BAC (5% of collisions, collision class 5).

Consistent with previous research, the findings concur that the most prevalent collisions occur when there are heightened opportunities for conflicts between road users, including busy urban roads [45], and during peak travel times when there are higher levels of traffic congestion [46]. Inclement weather conditions also play a role in collision risk, particularly precipitation [46], winter storms and icy roads [47] and impaired visibility conditions [48]. However, inclement conditions did not differentiate well between the collision classes in this study. A related analysis of the injury description for the present cohort found that weather conditions only played a direct role in a small number of collisions [33]. Moreover, while high temperatures are associated with elevated risk of motor vehicle collisions [49], these did not differ meaningfully between the collision classes. Other key factors that play an important role in the risk of road trauma include driver error, distraction or negligence—for instance, visual distractions (e.g., map reading or wandering gaze along the horizon or roadside), cognitive distractions (e.g., lost concentration or mind wandering), auditory distractions (e.g., a ringing cell phone), biomechanical distractions (e.g., leaning to manually adjust radio settings) [43] or speeding, drugs or alcohol [50]. Unfortunately, however, the presence of these experiences were not available in the present data.

There were not large differences in collision classes by day of the week. It appeared that most collisions occurred as people went about their regular work or social activities given the distribution of the collision classes at different times of day. The two classes of collisions with a higher prevalence of drivers with high levels of alcohol involved collisions where no other driver was at fault, predominantly in single-vehicle collisions that occurred in the evening and on the weekends. More than three quarters of the drivers injured in these collisions were younger males with an average age of 34 years. These findings are not surprising given that, in Australia, alcohol use is more prevalent in younger people and males [51], peaks in use on the weekends [52] and increases the risk of injury [53]. It should also be noted that the study excluded collisions in which the driver intended to harm themselves, which may include a range of other features, including substance use and serious mental health conditions and the presence of other life stressors.

People injured in the single-vehicle collisions (class 2) and collisions occurring predominantly at sunrise or sunset (class 4) had the best health and functional outcomes, whereas people injured in collisions that occurred in the late afternoon or evening when another was at fault (class 5) had the worst work outcomes. We suggest that these differences may be proxy indicators of other characteristics such as socioeconomic status if the sunrise/sunset collisions were predominantly for drivers commuting to and from work. Moreover, it is well known that there are negative long-term impacts when the injury occurred when another is at fault [54]. This interpretation is particularly plausible given that there were no differences in outcomes for people belonging to each of the collision classes when accounting for demographic, health and injury characteristics. Rather, people who were younger, male, with better pre-injury health and socioeconomic position, and lower injury severity had better recovery. In summary, the present findings highlight that although collision characteristics are associated with better or worse outcomes, these characteristics are probably proxy indicators of the driver’s demographic and health characteristics that have a stronger association with health and work outcomes.

### Study Strengths and Limitations

A major strength of the present study was the inclusion of population-level data for all hospital admissions for motor vehicle drivers who survived a serious injury and had a compensation claim within the Victorian road transport compensation scheme. Moreover, the linkage of trauma registry, insurance and meteorological data is novel and provided a robust dataset to examine collision characteristics. However, the present study has some limitations that should be considered. First, while the study only included major trauma cases, we excluded people if they died before transfer to hospital or within the first 12 months of injury. Therefore, the study does not characterise the nature of collisions resulting in less serious injuries, or for collisions that resulted in death. Outcomes could only be examined in 93% of the sample, with people who were completely lost to follow-up being younger, non-English speaking, had a lower socioeconomic position and disproportionately being injured in collisions where no other was at fault, and for drivers who had a high BAC reading. Data on some important characteristics known to be associated with road trauma risk and injury severity were not available, including licence type, vehicle make and model, vehicle insurance, road speed limits or travel speed, cell phone use, drug use or other driving infringements during the collision [12,42,43,44].

While the latent classes identified appear to reflect typical collisions, it should be noted that the results of any LCA are based on the researchers’ evaluation of model fit as well as their understanding of the substantive meaning of the results. Moreover, as this is a data-driven approach to identify the predominant classes of collisions, the findings may be specific to this cohort [37], and the findings should be replicated in other samples and settings. The characteristics of motor vehicle collisions may change over time in response to the implementation of road safety measures, which could not be captured in the present study, and may impact on the potential replication of the present findings. To counter the limitations of the LCA approach, we have fully disclosed the methods used to select the final class solution and to determine its reliability and validity in accordance with guidelines for reporting on latent trajectory studies [55].

## 5. Conclusions

In summary, this study identified five key latent classes of collisions that resulted in serious injury for Victorian drivers who had been admitted to hospital and survived following discharge. The collision classes varied in the time of day and the complexity of the collision, especially whether and how other vehicles were involved in the collision. The present findings highlight that using both environmental and person-focused variables can leverage the insights that can be gained from registry data. These methods could be extended to datasets that include collisions that cause less serious injuries as well as those resulting in death, in order to inform road safety strategies and campaigns to reduce the risk and impacts of road trauma at a population level.

## Figures and Tables

**Table 1:** Characteristics of the participants who were included in the study, and people who were completely lost to follow-up for EQ-5D, GOS-E or return to work outcomes analyses, *N* = 2735.

		Included (*n* = 2539)	Lost to Follow-Up (*n* = 196)	
		*n* (%)	*n* (%)	*p*-Value
Age (years)	15 to 24	496 (19.5)	45 (23.0)	<0.001
	25 to 34	404 (15.9)	60 (30.6)	
	35 to 44	415 (16.3)	31 (15.8)	
	45 to 54	338 (13.3)	13 (6.6)	
	55 to 64	306 (12.1)	16 (8.2)	
	65 to 74	263 (10.4)	20 (10.2)	
	75+	317 (12.5)	11 (5.6)	
Sex	Male	1607 (63.3)	136 (69.4)	0.087
	Female	932 (36.7)	60 (30.6)	
Preferred language, English ^a^	No	59 (2.9)	14 (8.8)	<0.001
	Yes	1981 (97.1)	145 (91.2)	
Residential area ^b^	Regional and remote	947 (37.7)	56 (30.3)	0.043
	Major cities	1564 (62.3)	129 (69.7)	
IRSAD (quintile) ^b^	1, highest disadvantage	522 (20.8)	62 (33.5)	0.002
	2	527 (21.0)	33 (17.8)	
	3	527 (21.0)	30 (16.2)	
	4	462 (18.4)	33 (17.8)	
	5, lowest disadvantage	473 (18.8)	27 (14.6)	
Education level ^c^	University	377 (15.7)	<5	<0.001
	Completed high school	365 (15.2)	<5	
	Advanced diploma	796 (33.1)	<5	
	Did not complete high school	868 (36.1)	187 (97.4)	
CCI conditions	Yes	1830 (72.1)	148 (75.5)	0.300
	No	709 (27.9)	48 (24.5)	
Blood alcohol ≥ 0.05 ^d^	No	1178 (85.1)	75 (70.8)	<0.001
	Yes	206 (14.9)	31 (29.2)	
Pre-injury mental health condition ^e^	No	2272 (90.2)	180 (91.8)	0.460
	Yes	246 (9.8)	16 (8.2)	
Pre-injury substance use condition ^e^	No	2271 (90.2)	165 (84.2)	0.008
	Yes	247 (9.8)	31 (15.8)	
Pre-injury disability ^f^	No	1918 (83.1)	<5	0.650
	Yes	389 (16.9)	<5	
Occupation skill level/status ^g^	Professionals	454 (18.1)	<5	0.600
	Trade/advanced clerical	412 (16.5)	<5	
	Intermediate	360 (14.4)	<5	
	Elementary/labourers	274 (11.0)	<5	
	Not working	864 (34.5)	<5	
	Studying	138 (5.5)	<5	
Fault attribution	Another at fault	443 (17.4)	17 (8.7)	<0.001
	Claim another at fault	125 (4.9)	6 (3.1)	
	No/deny other at fault	1432 (56.4)	111 (56.6)	
	Unknown if other at fault	539 (21.2)	62 (31.6)	
ISS (tertiles)	1 to 10	862 (34.0)	81 (41.3)	0.036
	11 to 17	952 (37.5)	74 (37.8)	
	18 to 75	725 (28.6)	41 (20.9)	
Injured body regions	Orthopaedic injuries	650 (25.6)	59 (30.1)	0.250
	Chest/abdominal injuries	993 (39.1)	65 (33.2)	
	Neurotrauma	311 (12.2)	21 (10.7)	
	Other	585 (23.0)	51 (26.0)	

Missing data: ^a^ *n* = 536, ^b^ *n* = 39, ^c^ *n* = 137, ^d^ *n* = 1245, ^e^ *n* = 21, ^f^ *n* = 427, ^g^ *n* = 225.

**Table 2:** Proportion of cases in each of the five classes who had the indicated characteristic.

	Class 1 (*n* = 663, 30.2%)	Class 2 (*n* = 600, 25.1%)	Class 3 (*n* = 711, 25.9%)	Class 4 (*n* = 365, 14.4%)	Class 5 (*n* = 128, *n* = 4.4%)
Another at fault	0.21	0.04	0.09	0.09	1.00
Multi-vehicle collision	1.00	0.03	0.40	0.52	1.00
Others seriously injured	0.21	0.10	0.14	0.14	0.38
BAC ≥ 0.05	0.02	0.12	0.36	0.16	0.00
Inclement weather	0.19	0.24	0.33	0.28	0.30
Regional/remote location	0.32	0.61	0.37	0.49	0.08
Time of week and day					
Weekend	0.26	0.25	0.40	0.25	0.23
Before sunrise	0.00	0.00	0.00	0.19	0.09
After sunrise	0.00	0.00	0.00	0.27	0.04
Daytime	1.00	1.00	0.00	0.00	0.00
Before sunset	0.00	0.00	0.00	0.29	0.18
After sunset	0.00	0.00	0.00	0.25	0.15
Evening	0.00	0.00	1.00	0.00	0.55

Abbreviations: BAC = blood alcohol concentration. *Notes*: Inclement weather: precipitation > 0.1 mm/h, gust > 20 km/h, visibility < 1 km.

**Table 3:** Association between motor vehicle collision classes and health, functional and work outcomes, adjusting for all covariates.

	EQ-5D Summary Score (*n* = 2532)	Independent Function (GOS-E; *n* = 2537)	Return to Work (*n* = 1529)
	Mean Difference, adj. (95%CI)	AOR (95%CI)	AOR (95%CI)
Collision class			
1	reference	1.00	1.00
2	0.02 (−0.01, 0.05)	1.06 (0.71, 1.58)	1.81 (0.91, 3.59)
3	0.00 (−0.02, 0.03)	1.09 (0.71, 1.67)	1.55 (0.77, 3.13)
4	0.02 (−0.01, 0.05)	1.09 (0.69, 1.73)	1.66 (0.79, 3.47)
5	−0.03 (−0.07, 0.01)	0.93 (0.45, 1.96)	0.37 (0.13, 1.05)
Age (years)			
15 to 24	reference	1.00	1.00
25 to 34	**−0.10 (−0.13, −0.07)**	**0.24 (0.15, 0.39)**	**0.10 (0.05, 0.19)**
35 to 44	**−0.11 (−0.14, −0.08)**	**0.21 (0.13, 0.34)**	**0.07 (0.04, 0.15)**
45 to 54	**−0.11 (−0.15, −0.08)**	**0.21 (0.12, 0.35)**	**0.15 (0.07, 0.29)**
55 to 64	**−0.08 (−0.12, −0.05)**	**0.32 (0.19, 0.55)**	**0.09 (0.04, 0.20)**
65 to 74	−0.04 (−0.08, 0.00)	0.66 (0.36, 1.21)	**0.05 (0.01, 0.15)**
75+	−0.03 (−0.07, 0.01)	0.55 (0.29, 1.02)	**0.10 (0.02, 0.49)**
Sex			
Male	reference	1.00	1.00
Female	**−0.04 (−0.05, −0.02)**	0.86 (0.64, 1.14)	0.66 (0.42, 1.03)
Preferred language, English			
No	reference	1.00	1.00
Yes	0.04 (−0.01, 0.10)	1.53 (0.60, 3.89)	3.15 (0.61, 16.17)
Residential area			
Regional and remote areas	reference	1.00	1.00
Major cities	−0.02 (−0.04, 0.00)	0.78 (0.57, 1.06)	0.76 (0.48, 1.18)
IRSAD (quintile)			
1, highest disadvantage	reference	1.00	1.00
2	**0.03 (0.01, 0.06)**	1.28 (0.84, 1.95)	**2.71 (1.49, 4.91)**
3	**0.03 (0.01, 0.06)**	1.23 (0.82, 1.85)	**1.90 (1.04, 3.45)**
4	**0.04 (0.01, 0.07)**	**1.70 (1.10, 2.62)**	**3.23 (1.68, 6.20)**
5, highest advantage	**0.06 (0.03, 0.09)**	**1.95 (1.24, 3.08)**	**5.39 (2.54, 11.41)**
Occupation skill level/status			
Elementary/labourers	reference	1.00	1.00
Intermediate	0.02 (−0.01, 0.05)	**2.14 (1.21, 3.79)**	**4.16 (2.18, 7.92)**
Trade/advanced clerical	0.01 (−0.02, 0.04)	**1.98 (1.14, 3.44)**	**3.99 (2.08, 7.66)**
Professionals	0.04 (0.00, 0.07)	**2.57 (1.48, 4.45)**	**20.20 (9.36, 43.60)**
Not working	−0.03 (−0.07, 0.00)	**8.21 (4.72, 14.28)**	n/a
Education level			
Did not complete high school	reference	1.00	1.00
Advanced diploma	0.03 (0.00, 0.05)	1.03 (0.74, 1.44)	1.67 (0.98, 2.83)
Completed high school	**0.03 (0.01, 0.06)**	1.50 (0.99, 2.29)	**3.72 (1.86, 7.44)**
University	**0.05 (0.03, 0.08)**	**2.31 (1.51, 3.54)**	**8.11 (3.79, 17.35)**
CCI conditions			
Yes	reference	1.00	1.00
No	**0.03 (0.01, 0.05)**	**1.97 (1.39, 2.78)**	**6.45 (3.53, 11.77)**
Pre-injury mental health condition			
No	reference	1.00	1.00
Yes	0.00 (−0.03, 0.03)	0.67 (0.41, 1.10)	0.86 (0.41, 1.80)
Pre-injury substance use condition			
No	reference	1.00	1.00
Yes	0.00 (−0.04, 0.03)	1.32 (0.81, 2.15)	1.24 (0.55, 2.82)
Pre-injury disability			
No	reference	1.00	1.00
Yes	**−0.05 (−0.08, −0.03)**	**0.41 (0.27, 0.61)**	**0.44 (0.21, 0.93)**
Fault attribution			
Another at fault	reference	1.00	1.00
Claim another at fault	−0.03 (−0.07, 0.02)	0.93 (0.47, 1.88)	0.26 (0.10, 0.70)
No other at fault	0.02 (−0.01, 0.05)	**1.97 (1.25, 3.12)**	0.72 (0.37, 1.37)
Deny another at fault	0.00 (−0.07, 0.06)	1.19 (0.41, 3.43)	1.57 (0.38, 6.40)
Unknown if another at fault	0.00 (−0.03, 0.03)	1.56 (0.94, 2.59)	0.93 (0.46, 1.88)
ISS (tertiles)			
1–10	reference	1.00	1.00
11–17	**−0.04 (−0.06, −0.02)**	**0.46 (0.31, 0.69)**	**0.23 (0.13, 0.41)**
18–75	**−0.10 (−0.13, −0.07)**	**0.15 (0.09, 0.25)**	**0.04 (0.02, 0.09)**
Injured body regions			
Orthopaedic injuries only	reference	1.00	1.00
Chest/abdominal injuries	**0.06 (0.03, 0.08)**	**3.30 (2.06, 5.29)**	**5.70 (2.77, 11.73)**
Head injury	**0.10 (0.07, 0.14)**	**4.48 (2.45, 8.17)**	**13.07 (5.22, 32.73)**
Spinal cord injury	**−0.20 (−0.28, −0.12)**	0.05 (0.00, 0.75)	**0.13 (0.02, 0.95)**
Other/multi-trauma injuries	0.01 (−0.01, 0.04)	1.37 (0.92, 2.03)	**1.95 (1.11, 3.43)**
Months post-injury			
6 months	reference	1.00	1.00
12 months	**0.02 (0.01, 0.03)**	**1.57 (1.31, 1.89)**	**2.52 (1.94, 3.27)**
24 months	0.01 (0.00, 0.02)	**1.97 (1.60, 2.41)**	**6.45 (3.53, 11.77)**

Notes: AOR = adjusted odds ratio. Significant associations are emphasised in bold. The descriptive statistics and unadjusted estimates are reported in Tables S4–S6.

## Data Availability

The authors do not have approval from the data custodians at the VSTR or the TAC to publish the original data. Any external parties wishing to access the data can do so through discussion with the authors, by obtaining their own ethics or relevant equivalent approvals, and securing approval from the data custodians.

## Supplementary Materials

The following are available online at https://www.mdpi.com/article/10.3390/ijerph182111380/s1, Table S1: Model fit parameters with each increase in the number of classes, Table S2: Comparison of latent class characteristics, *N* = 2467, Table S3: Five-class solution in random sample 1 and 2 without covariates, Table S4: Five-class solution in random sample 1 and 2 with covariates of age and sex, Table S5: EQ-5D summary score descriptive statistics and estimated mean differences, *N* = 2532, Table S6: Return to work descriptive statistics and estimated mean differences, *N* = 1529, Table S7: GOS-E independent function outcome descriptive statistics and estimated mean differences, *N* = 2537.
