# Supplementary material for: Driver, Collision and Meteorological Characteristics of Motor Vehicle Collisions among Road Trauma Survivors
Source: Int J Environ Res Public Health. 2021 Oct 29;18(21):11380. doi: 10.3390/ijerph182111380 (PMC8583338; doi:10.3390/ijerph182111380)
Supplement: Supplementary file 1 [file ijerph-18-11380-s001.zip › ijerph-1425585-supplementary.pdf]

Supplementary Materials

Table S1. Model fit parameters with each increase in the number of classes.

	Number of Classes					
	**1**	**2**	**3**	**4**	**5**	**6**
Iterations for convergence	16	30	93	335	413	1389
Adjusted BIC	5192.39	2892.28	1993.25	1756.07	1698.40	1746.44
BIC	5233.70	2978.07	2123.52	1930.82	1917.64	2010.16
AIC	5156.82	2818.40	1881.05	1605.55	1509.58	1519.31
Entropy R^2	1.00	0.91	0.92	0.83	0.79	0.76
Log-likelihood of fixed parameters	−14662.95	−13479.74	−12997.07	−12845.32	−12783.33	−12774.20
Proportion (SE) of cases per class						
Class 1	1.00 (1.00)	0.56 (0.01)	0.56 (0.01)	0.32 (0.03)	0.31 (0.03)	0.13 (0.04)
Class 2		0.44 (0.01)	0.16 (0.01)	0.24 (0.03)	0.25 (0.03)	0.15 (0.04)
Class 3			0.28 (0.01)	0.28 (0.01)	0.18 (0.02)	0.18 (0.02)
Class 4				0.16 (0.01)	0.16 (0.01)	0.11 (0.02)
Class 5					0.11 (0.02)	0.16 (0.01)
Class 6						0.28 (0.03)

Table S2. Comparison of latent class characteristics, N = 2467.

		Class 1 (n = 663) N (%)	Class 2 (n = 600) N (%)	Class 3 (n = 711) N (%)	Class 4 (n = 365) N (%)	Class 5 (n = 128) N (%)	*p*-Value
Fault attribution	Another at fault	115 (17.3)	15 (2.5)	40 (5.6)	22 (6.0)	109 (85.2)	<0.001
	Claim another at fault	26 (3.9)	12 (2.0)	23 (3.2)	11 (3.0)	19 (14.8)	
	No other at fault	362 (54.6)	386 (64.3)	450 (63.3)	233 (63.8)	0 (0.0)	
	Deny another at fault	9 (1.4)	24 (4.0)	12 (1.7)	7 (1.9)	0 (0.0)	
	Unknown if another was at fault	151 (22.8)	163 (27.2)	186 (26.2)	92 (25.2)	0 (0.0)	
Age (years)	15 to 24	38 (5.7)	96 (16.0)	238 (33.5)	94 (25.8)	14 (10.9)	<0.001
	25 to 34	59 (8.9)	85 (14.2)	187 (26.3)	50 (13.7)	21 (16.4)	
	35 to 44	64 (9.7)	81 (13.5)	131 (18.4)	82 (22.5)	30 (23.4)	
	45 to 54	83 (12.5)	86 (14.3)	76 (10.7)	48 (13.2)	25 (19.5)	
	55 to 64	104 (15.7)	98 (16.3)	39 (5.5)	40 (11.0)	20 (15.6)	
	65 to 74	128 (19.3)	70 (11.7)	26 (3.7)	24 (6.6)	16 (12.5)	
	75+	187 (28.2)	84 (14.0)	14 (2.0)	27 (7.4)	<5	
Sex	Male	293 (44.2)	388 (64.7)	545 (76.7)	245 (67.1)	81 (63.3)	<0.001
	Female	370 (55.8)	212 (35.3)	166 (23.3)	120 (32.9)	47 (36.7)	
Preferred Language, English	No	22 (4.2)	13 (2.7)	13 (2.3)	11 (3.7)	<5	0.23
	Yes	504 (95.8)	476 (97.3)	557 (97.7)	289 (96.3)	100 (99.0)	
Residential Area	Regional and remote areas	200 (30.4)	310 (52.5)	230 (32.9)	165 (45.7)	17 (13.6)	<0.001
	Major cities	458 (69.6)	280 (47.5)	469 (67.1)	196 (54.3)	108 (86.4)	
IRSAD (quintile)	1, highest level of disadvantage	136 (20.7)	124 (21.0)	172 (24.6)	75 (20.8)	19 (15.2)	0.007
	2	113 (17.2)	139 (23.6)	150 (21.5)	91 (25.2)	22 (17.6)	
	3	126 (19.1)	117 (19.8)	144 (20.6)	80 (22.2)	30 (24.0)	
	4	136 (20.7)	111 (18.8)	120 (17.2)	58 (16.1)	24 (19.2)	
	5, highest level of advantage	147 (22.3)	99 (16.8)	113 (16.2)	57 (15.8)	30 (24.0)	
Education level	Did not complete high school	281 (45.2)	238 (42.0)	284 (41.9)	142 (40.6)	29 (23.4)	<0.001
	Advanced diploma	166 (26.7)	166 (29.3)	223 (32.9)	101 (28.9)	54 (43.5)	
	Completed high school	76 (12.2)	79 (13.9)	99 (14.6)	53 (15.1)	20 (16.1)	
	University	99 (15.9)	84 (14.8)	72 (10.6)	54 (15.4)	21 (16.9)	

		Class 1 (n = 663) N (%)	Class 2 (n = 600) N (%)	Class 3 (n = 711) N (%)	Class 4 (n = 365) N (%)	Class 5 (n = 128) N (%)	*p*-Value
Occupation skill level/status	Not working	346 (56.1)	205 (37.1)	143 (22.3)	96 (28.8)	22 (18.3)	<0.001
	Elementary/labourers	48 (7.8)	61 (11.0)	81 (12.7)	45 (13.5)	15 (12.5)	
	Intermediate	63 (10.2)	66 (11.9)	119 (18.6)	52 (15.6)	29 (24.2)	
	Trade/ advanced clerical	36 (5.8)	90 (16.3)	156 (24.4)	54 (16.2)	24 (20.0)	
	Associate professionals	29 (4.7)	31 (5.6)	32 (5.0)	17 (5.1)	8 (6.7)	
	Managers/ professionals	73 (11.8)	71 (12.8)	65 (10.2)	49 (14.7)	15 (12.5)	
	Studying	22 (3.6)	29 (5.2)	44 (6.9)	20 (6.0)	7 (5.8)	
CCI conditions	No	542 (82.2)	479 (80.1)	498 (70.2)	301 (82.5)	106 (82.8)	<0.001
	Yes	117 (17.8)	119 (19.9)	211 (29.8)	64 (17.5)	22 (17.2)	
Pre-injury mental health condition	No	591 (90.1)	539 (90.7)	622 (88.0)	328 (90.9)	120 (93.8)	0.21
	Yes	65 (9.9)	55 (9.3)	85 (12.0)	33 (9.1)	8 (6.3)	
Pre-injury substance use condition	No	631 (96.2)	551 (92.8)	551 (77.9)	323 (89.5)	124 (96.9)	<0.001
	Yes	25 (3.8)	43 (7.2)	156 (22.1)	38 (10.5)	4 (3.1)	
Pre-injury disability	No	445 (77.1)	412 (81.3)	486 (85.9)	260 (83.6)	105 (91.3)	<0.001
	Yes	132 (22.9)	95 (18.7)	80 (14.1)	51 (16.4)	10 (8.7)	
ISS (tertiles)	1–10	234 (35.3)	214 (35.7)	231 (32.5)	128 (35.1)	46 (35.9)	0.68
	11–17	230 (34.7)	226 (37.7)	277 (39.0)	142 (38.9)	50 (39.1)	
	18–75	199 (30.0)	160 (26.7)	203 (28.6)	95 (26.0)	32 (25.0)	
Injured body regions	Orthopaedic injuries only	166 (25.0)	179 (29.8)	164 (23.1)	98 (26.8)	33 (25.8)	0.020
	Chest/abdominal (with/without additional injuries)	269 (40.6)	233 (38.8)	260 (36.6)	131 (35.9)	53 (41.4)	
	Head injury	75 (11.3)	68 (11.3)	92 (12.9)	34 (9.3)	10 (7.8)	
	Spinal cord injury	6 (0.9)	7 (1.2)	12 (1.7)	<5	0 (0.0)	
	Other/multi-trauma injuries	147 (22.2)	113 (18.8)	183 (25.7)	101 (27.7)	32 (25.0)	

Notes: Values between 1–5 are suppressed.

Table S3. Five class solution in random sample 1 and 2 without covariates.

	**Another at Fault**		**Multi-Vehicle Collision**		**Others Seriously Injured**		**BAC > = 0.05**		**Injury Location Regional/Remote**		**Inclement Weather**		**Weekend**		**before Sunrise**		**after Sunrise**		**Daytime**		**before Sunset**		**after Sunset**		**Evening**	
	**S1**	**S2**	**S1**	**S2**	**S1**	**S2**	**S1**	**S2**	**S1**	**S2**	**S1**	**S2**	**S1**	**S2**	**S1**	**S2**	**S1**	**S2**	**S1**	**S2**	**S1**	**S2**	**S1**	**S2**	**S1**	**S2**
Class 1	0.47	0.33	1.00	1.00	0.31	0.28	0.00	0.04	0.23	0.31	0.21	0.23	0.25	0.25	0.01	0.01	0.00	0.00	1.00	1.00	0.00	0.00	0.00	0.00	0.00	0.00
Class 2	0.03	0.05	0.21	0.00	0.08	0.11	0.07	0.12	0.61	0.63	0.22	0.23	0.25	0.25	0.03	0.01	0.00	0.00	1.00	1.00	0.00	0.00	0.00	0.00	0.00	0.00
Class 3	0.00	0.08	0.18	0.01	0.04	0.07	0.45	0.36	0.48	0.50	0.28	0.33	0.32	0.45	0.00	0.00	0.00	0.00	0.00	0.00	0.00	0.00	0.00	0.00	1.00	1.00
Class 4 *	0.18	0.25	0.60	0.60	0.16	0.19	0.17	0.11	0.43	0.44	0.25	0.31	0.23	0.24	0.16	0.15	0.27	0.24	0.00	0.00	0.35	0.29	0.22	0.32	0.00	0.00
Class 5 *	0.39	0.34	0.86	0.99	0.32	0.26	0.27	0.25	0.09	0.19	0.36	0.29	0.44	0.33	0.00	0.00	0.00	0.00	0.00	0.00	0.00	0.00	0.00	0.00	1.00	1.00

Abbreviations: S = sample. * Class 4 was actually Class 5 for Sample 2, and vice versa.

Table S4. Five class solution in random sample 1 and 2 with covariates of age and sex.

	**Another at Fault**		**Multi-Vehicle Collision**		**Others Seriously Injured**		**BAC > = 0.05**		**Injury Location Regional/Remote**		**Inclement Weather**		**Weekend**		**before Sunrise**		**after sunrise**		**Daytime**		**before Sunset**		**after Sunset**		**Evening**	
	**S1**	**S2**	**S1**	**S2**	**S1**	**S2**	**S1**	**S2**	**S1**	**S2**	**S1**	**S2**	**S1**	**S2**	**S1**	**S2**	**S1**	**S2**	**S1**	**S2**	**S1**	**S2**	**S1**	**S2**	**S1**	**S2**
Class 1	0.42	0.37	1.00	1.00	0.28	0.31	0.00	0.02	0.31	0.28	0.20	0.21	0.25	0.27	0.01	0.00	0.00	0.00	1.00	1.00	0.00	0.00	0.00	0.00	0.00	0.00
Class 2	0.04	0.05	0.10	0.12	0.08	0.10	0.08	0.16	0.60	0.63	0.22	0.26	0.23	0.24	0.00	0.00	0.00	0.00	1.00	1.00	0.00	0.00	0.00	0.00	0.00	0.00
Class 3	0.16	0.07	0.51	0.11	0.18	0.07	0.36	0.36	0.31	0.50	0.33	0.33	0.38	0.43	0.00	0.00	0.00	0.00	0.00	0.00	0.00	0.00	0.00	0.00	1.00	1.00
Class 4 *	0.08	0.25	0.42	0.63	0.10	0.20	0.13	0.10	0.55	0.44	0.34	0.31	0.27	0.24	0.29	0.17	0.36	0.23	0.00	0.00	0.24	0.28	0.10	0.31	0.00	0.00
Class 5 *	0.37	0.41	0.93	1.00	0.30	0.29	0.23	0.18	0.15	0.14	0.07	0.31	0.18	0.31	0.00	0.00	0.00	0.00	0.00	0.00	0.55	0.00	0.45	0.00	0.00	**1.00**

Abbreviations: S = sample. * Note that Class 4 in sample 1 had the characteristics of Class 5 for Sample 2, and vice versa.

Table S5. EQ-5D summary score descriptive statistics and estimated mean differences, N = 2532.

		EQ-5D Summary Score				
		6 Months m (sd)	12 Months m (sd)	24 Months m (sd)	**Mean Difference (95%CI)**	**Mean Difference, Adjusted (95%CI)**
MVA, latent class	1	0.63 (0.26)	0.67 (0.24)	0.65 (0.26)	reference	reference
	2	0.69 (0.24)	0.7 (0.25)	0.69 (0.26)	**0.04 (0.01, 0.06)**	0.02 (−0.01, 0.05)
	3	0.67 (0.25)	0.69 (0.26)	0.68 (0.27)	**0.03 (0.00, 0.05)**	0.00 (−0.02, 0.03)
	4	0.68 (0.23)	0.69 (0.25)	0.70 (0.26)	**0.04 (0.01, 0.07)**	0.02 (−0.01, 0.05)
	5	0.60 (0.23)	0.63 (0.26)	0.62 (0.24)	−0.02 (−0.06, 0.02)	−0.03 (−0.07, 0.01)
Age (years)	15 to 24	0.74 (0.22)	0.76 (0.23)	0.78 (0.23)	reference	reference
	25 to 34	0.64 (0.24)	0.66 (0.26)	0.67 (0.27)	**−0.10 (−0.13, −0.07)**	**−0.10 (−0.13, −0.07)**
	35 to 44	0.62 (0.26)	0.65 (0.25)	0.64 (0.26)	**−0.12 (−0.15, −0.09)**	**−0.11 (−0.14, −0.08)**
	45 to 54	0.61 (0.26)	0.63 (0.27)	0.63 (0.27)	**−0.13 (−0.16, −0.10)**	**−0.11 (−0.15, −0.08)**
	55 to 64	0.65 (0.24)	0.67 (0.24)	0.66 (0.25)	**−0.09 (−0.12, −0.06)**	**−0.08 (−0.12, −0.05)**
	65 to 74	0.67 (0.24)	0.69 (0.24)	0.65 (0.26)	**−0.09 (−0.12, −0.05)**	−0.04 (−0.08, 0.00)
	75+	0.67 (0.24)	0.68 (0.23)	0.64 (0.25)	**−0.09 (−0.12, −0.06)**	−0.03 (−0.07, 0.01)
Sex	Male	0.67 (0.25)	0.69 (0.25)	0.68 (0.26)	reference	reference
	Female	0.64 (0.24)	0.66 (0.25)	0.66 (0.25)	**−0.03 (−0.05, −0.01)**	**−0.04 (−0.05, −0.02)**
Preferred Language, English	No	0.59 (0.27)	0.59 (0.29)	0.57 (0.27)	reference	reference
	Yes	0.67 (0.25)	0.69 (0.25)	0.68 (0.26)	**0.06 (0.01, 0.12)**	0.04 (−0.01, 0.10)
Residential Area	Regional and remote areas	0.67 (0.24)	0.69 (0.24)	0.69 (0.25)	reference	reference
	Major cities	0.65 (0.25)	0.68 (0.26)	0.66 (0.27)	−0.01 (−0.03, 0.00)	−0.02 (−0.04, 0.00)
IRSAD (quintile)	1, highest level of disadvantage	0.63 (0.26)	0.65 (0.25)	0.64 (0.26)	reference	reference
	2	0.65 (0.25)	0.70 (0.23)	0.67 (0.26)	**0.03 (0.00, 0.06)**	**0.03 (0.01, 0.06)**
	3	0.67 (0.25)	0.66 (0.27)	0.68 (0.25)	**0.03 (0.00, 0.06)**	**0.03 (0.01, 0.06)**
	4	0.66 (0.25)	0.69 (0.25)	0.68 (0.27)	**0.03 (0.01, 0.06)**	**0.04 (0.01, 0.07)**
	5, highest level of advantage	0.69 (0.23)	0.71 (0.25)	0.69 (0.26)	**0.05 (0.03, 0.08)**	**0.06 (0.03, 0.09)**
Education level	Did not complete high school	0.64 (0.26)	0.66 (0.26)	0.64 (0.27)	reference	reference
	Advanced diploma	0.67 (0.24)	0.68 (0.23)	0.69 (0.25)	**0.03 (0.01, 0.06)**	**0.03 (0.00, 0.05)**
	Completed high school	0.69 (0.25)	0.7 (0.26)	0.72 (0.24)	**0.05 (0.02, 0.08)**	**0.03 (0.01, 0.06)**

		EQ-5D Summary Score				
		6 Months m (sd)	12 Months m (sd)	24 Months m (sd)	Mean Difference (95%CI)	Mean Difference, Adjusted (95%CI)
	University	0.69 (0.22)	0.72 (0.24)	0.72 (0.26)	**0.06 (0.04, 0.09)**	**0.05 (0.03, 0.08)**
CCI conditions	Yes	0.64 (0.26)	0.65 (0.26)	0.63 (0.26)	reference	reference
	No	0.67 (0.24)	0.69 (0.24)	0.69 (0.26)	**0.04 (0.02, 0.06)**	**0.03 (0.01, 0.05)**
Pre-injury mental health condition	No	0.66 (0.25)	0.68 (0.25)	0.68 (0.26)	reference	reference
	Yes	0.64 (0.26)	0.66 (0.27)	0.64 (0.28)	−0.03 (−0.06, 0.00)	0.00 (−0.03, 0.03)
Pre-injury substance use condition	No	0.66 (0.25)	0.68 (0.25)	0.68 (0.26)	reference	reference
	Yes	0.64 (0.25)	0.66 (0.25)	0.64 (0.28)	−0.03 (−0.06, 0.00)	0.00 (−0.04, 0.03)
Pre-injury disability	No	0.68 (0.25)	0.7 (0.25)	0.69 (0.26)	reference	reference
	Yes	0.60 (0.25)	0.62 (0.25)	0.61 (0.26)	**−0.08 (−0.10, −0.06)**	**-0.05 (−0.08, −0.03)**
Occupation skill level/status	Elementary/labourers	0.66 (0.26)	0.67 (0.26)	0.67 (0.25)	reference	reference
	Intermediate	0.66 (0.25)	0.68 (0.26)	0.68 (0.26)	0.01 (−0.03, 0.04)	0.02 (−0.01, 0.05)
	Trade/ advanced clerical	0.69 (0.24)	0.71 (0.23)	0.73 (0.24)	**0.04 (0.00, 0.07)**	0.01 (−0.02, 0.04)
	Professionals	0.69 (0.23)	0.72 (0.22)	0.72 (0.23)	**0.04 (0.00, 0.07)**	**0.04 (0.00, 0.07)**
	Not working	0.63 (0.25)	0.64 (0.26)	0.62 (0.27)	**−0.04 (−0.07, −0.01)**	−0.03 (−0.07, 0.00)
	Studying	0.69 (0.23)	0.73 (0.26)	0.74 (0.26)	**0.05 (0.01, 0.09)**	0.00 (−0.04, 0.04)
Fault attribution	Another at fault	0.63 (0.24)	0.66 (0.24)	0.65 (0.23)	reference	reference
	Claim another at fault	0.62 (0.26)	0.65 (0.25)	0.64 (0.27)	−0.01 (−0.06, 0.03)	−0.03 (−0.07, 0.02)
	No other at fault	0.68 (0.24)	0.70 (0.25)	0.69 (0.26)	0.04 (0.01, 0.06)	0.02 (−0.01, 0.05)
	Deny another at fault	0.67 (0.24)	0.65 (0.26)	0.70 (0.22)	0.01 (−0.05, 0.08)	0.00 (−0.07, 0.06)
	Unknown if another was at fault	0.65 (0.26)	0.67 (0.27)	0.66 (0.27)	0.01 (−0.02, 0.04)	0.00 (−0.03, 0.03)
ISS (tertiles)	1–10	0.68 (0.24)	0.69 (0.24)	0.70 (0.24)	reference	reference
	11–17	0.67 (0.24)	0.69 (0.25)	0.68 (0.26)	−0.01 (−0.03, 0.01)	**−0.04 (−0.06, −0.02)**
	18–75	0.62 (0.26)	0.66 (0.26)	0.64 (0.28)	**−0.05 (−0.07, −0.03)**	**−0.10 (−0.13, −0.07)**
Injured body regions	Orthopaedic injuries only	0.66 (0.25)	0.69 (0.23)	0.69 (0.24)	reference	reference
	Chest/abdominal (with/without additional injuries)	0.66 (0.25)	0.67 (0.26)	0.66 (0.27)	−0.01 (−0.03, 0.01)	**0.06 (0.03, 0.08)**
	Head injury	0.70 (0.24)	0.72 (0.23)	0.70 (0.25)	**0.03 (0.00, 0.06)**	**0.10 (0.07, 0.14)**
	Spinal cord injury	0.32 (0.32)	0.44 (0.33)	0.45 (0.33)	**−0.26 (−0.34, −0.17)**	**−0.20 (−0.28, −0.12)**
	Other/multi-trauma injuries	0.67 (0.24)	0.68 (0.26)	0.67 (0.26)	0.00 (−0.03, 0.02)	0.01 (−0.01, 0.04)

		EQ-5D Summary Score				
		6 Months m (sd)	12 Months m (sd)	24 Months m (sd)	Mean Difference (95%CI)	Mean Difference, Adjusted (95%CI)
Months post-injury	6 months	0.66 (0.25)			reference	reference
	12 months		0.68 (0.25)		**0.02 (0.01, 0.03)**	**0.02 (0.01, 0.03)**
	24 months			0.67 (0.26)	**0.01 (0.00, 0.02)**	**0.01 (0.00, 0.02)**

Notes: Missing data were imputed for the following covariates: latent class (253 cases), preferred language (498 cases), residential area and IRSAD (27 cases), education level (132 cases), occupation group (36 cases), preinjury mental health and substance use condition (21 cases), preinjury disability (228 cases). Significant associations are emphasised in bold.

Table S6. Return to work descriptive statistics and estimated mean differences, N = 1529.

		Return to Work				
		6 Months n (%)	12 Months n (%)	24 Months n (%)	OR (95%CI)	AOR (95%CI)
MVA, latent class	1	113 (48.9)	141 (60.0)	129 (60.8)	1.00	1.00
	2	174 (60.0)	192 (65.5)	180 (65.9)	**2.75 (1.33, 5.69)**	1.81 (0.91, 3.59)
	3	229 (56.3)	241 (61.5)	218 (63.9)	1.81 (0.91, 3.60)	1.55 (0.77, 3.13)
	4	111 (53.9)	126 (64.0)	112 (67.5)	**2.40 (1.10, 5.23)**	1.66 (0.79, 3.47)
	5	33 (37.5)	48 (52.2)	42 (53.2)	0.49 (0.18, 1.33)	0.37 (0.13, 1.05)
Age (years)	15 to 24	206 (65.2)	233 (73.0)	217 (78.3)	1.00	1.00
	25 to 34	127 (48.3)	149 (58.2)	138 (60.5)	**0.09 (0.05, 0.20)**	**0.10 (0.05, 0.19)**
	35 to 44	143 (48.5)	158 (55.2)	137 (58.1)	**0.07 (0.03, 0.15)**	**0.07 (0.04, 0.15)**
	45 to 54	120 (50.2)	137 (58.5)	135 (61.1)	**0.11 (0.05, 0.22)**	**0.15 (0.07, 0.29)**
	55 to 64	108 (54.8)	116 (59.8)	102 (58.3)	**0.12 (0.06, 0.27)**	**0.09 (0.04, 0.20)**
	65 to 74	31 (55.4)	34 (59.6)	27 (52.9)	**0.09 (0.03, 0.29)**	**0.05 (0.01, 0.15)**
	75+	11 (52.4)	15 (62.5)	14 (58.3)	**0.13 (0.03, 0.64)**	**0.10 (0.02, 0.49)**
Sex	Male	513 (53.2)	582 (60.9)	536 (64.0)	1.00	1.00
	Female	233 (55.1)	260 (62.8)	234 (62.6)	1.19 (0.79, 1.79)	0.66 (0.42, 1.03)
Preferred Language, English	No	9 (45.0)	9 (45.0)	9 (47.4)	1.00	1.00
	Yes	604 (55.0)	672 (62.4)	607 (64.9)	4.89 (0.81, 29.45)	3.15 (0.61, 16.17)
Residential Area	Regional and remote areas	314 (54.0)	355 (62.1)	332 (64.6)	1.00	1.00
	Major cities	426 (53.9)	476 (60.9)	431 (62.7)	0.85 (.057, 1.28)	0.76 (0.48, 1.18)
IRSAD (quintile)	1, highest level of disadvantage	130 (46.9)	147 (54.9)	136 (54.0)	1.00	1.00
	2	166 (51.4)	192 (60.6)	180 (64.3)	**2.44 (1.30, 4.58)**	**2.71 (1.49, 4.91)**

		Return to Work				
		6 Months n (%)	12 Months n (%)	24 Months n (%)	OR (95%CI)	AOR (95%CI)
	3	154 (51.2)	181 (59.9)	172 (66.2)	**2.53 (1.33, 4.81)**	**1.90 (1.04, 3.45)**
	4	148 (59.7)	163 (64.7)	136 (64.5)	**4.10 (2.06, 8.16)**	**3.23 (1.68, 6.20)**
	5, highest level of advantage	142 (63.4)	148 (68.8)	139 (70.2)	**8.94 (3.96, 20.18)**	**5.39 (2.54, 11.41)**
Education level	Did not complete high school	187 (47.1)	214 (54.2)	194 (55.9)	1.00	1.00
	Advanced diploma	258 (49.0)	312 (60.2)	307 (66.0)	**2.53 (1.41, 4.53)**	1.67 (0.98, 2.83)
	Completed high school	120 (62.8)	128 (68.4)	99 (66.0)	**6.99 (3.27, 14.95)**	**3.72 (1.86, 7.44)**
	University	165 (71.7)	171 (77.0)	153 (75.4)	**23.27 (9.48, 57.13)**	**8.11 (3.79, 17.35)**
CCI conditions	Yes	156 (44.1)	168 (47.7)	155 (48.9)	1.00	1.00
	No	590 (57.1)	674 (66.2)	615 (68.7)	**11.10 (5.67, 21.72)**	**6.45 (3.53, 11.77)**
Pre-injury mental health condition	No	687 (54.9)	770 (62.3)	704 (64.2)	1.00	1.00
	Yes	53 (42.4)	64 (51.6)	59 (55.7)	**0.28 (0.13, 0.57)**	0.86 (0.41, 1.80)
Pre-injury substance use condition	No	679 (54.2)	767 (62.0)	700 (64.7)	1.00	1.00
	Yes	61 (49.2)	67 (55.4)	63 (52.5)	**0.20 (0.09, 0.45)**	1.24 (0.55, 2.82)
Pre-injury disability	No	689 (54.6)	730 (62.7)	665 (65.0)	1.00	1.00
	Yes	52 (45.6)	60 (54.5)	50 (54.3)	**0.24 (0.11, 0.53)**	**0.44 (0.21, 0.93)**
Occupation skill level/status	Elementary/labourers	101 (41.2)	116 (47.2)	107 (48.0)	1.00	1.00
	Intermediate	160 (48.9)	184 (56.6)	166 (59.9)	**3.87 (1.93, 7.78)**	**4.16 (2.18, 7.92)**
	Trade/ advanced clerical	190 (52.1)	233 (63.1)	221 (69.5)	**9.07 (4.19, 19.62)**	**3.99 (2.08, 7.66)**
	Professionals	279 (66.4)	296 (73.8)	268 (72.4)	**35.67 (14.21, 89.58)**	**20.20 (9.36, 43.60)**
Fault attribution	Another at fault	139 (48.6)	168 (59.6)	156 (61.4)	1.00	1.00
	Claim another at fault	31 (47.7)	38 (55.1)	39 (60.9)	0.44 (0.16, 1.18)	0.26 (0.10, 0.70)
	No other at fault	396 (55.7)	431 (62.0)	385 (64.0)	1.61 (0.97, 2.65)	0.72 (0.37, 1.37)
	Deny another at fault	16 (61.5)	19 (73.1)	21 (91.3)	**6.09 (1.61, 23.00)**	1.57 (0.38, 6.40)
	Unknown if another was at fault	164 (54.8)	186 (62.4)	169 (62.8)	1.60 (0.89, 2.86)	0.93 (0.46, 1.88)
ISS (tertiles)	1–10	292 (61.1)	315 (66.7)	248 (67.2)	1.00	1.00
	11–17	277 (55.1)	319 (63.9)	308 (67.2)	**0.60 (0.39, 0.93)**	**0.23 (0.13, 0.41)**
	18–75	177 (43.6)	208 (52.1)	214 (55.6)	**0.11 (0.06, 0.21)**	**0.04 (0.02, 0.09)**
Injured body regions	Orthopaedic injuries only	233 (57.11)	267 (65.60)	226 (66.86)	1.00	1.00
	Chest/abdominal (with/without additional injuries)	283 (52.2)	322 (60.3)	315 (61.6)	**0.55 (0.34, 0.90)**	**5.70 (2.77, 11.73)**
	head injury	96 (54.55)	113 (63.84)	117 (69.64)	0.87 (0.46, 1.65)	**13.07 (5.22, 32.73)**

		Return to Work				
		6 Months	12 Months	24 Months		
		n (%)	n (%)	n (%)	OR (95%CI)	AOR (95%CI)
	SCI	4 (21.05)	5 (26.32)	3 (18.75)	**0.01 (0.00, 0.03)**	**0.13 (0.02, 0.95)**
	other/multi	202 (54.74)	229 (63.43)	194 (67.13)	0.85 (0.50, 1.44)	**1.95 (1.11, 3.43)**
Months post-injury	6 months	746 (53.8)			1.00	1.00
	12 months		842 (61.5)		**2.43 (1.90, 3.10)**	**2.52 (1.94, 3.27)**
	24 months			770 (63.5)	**3.31 (2.43, 4.50)**	**3.52 (2.53, 4.89)**

Notes: Missing data were imputed for the following covariates: latent class (180 cases), preferred language (304 cases), residential area and IRSAD (18 cases), education level (57 cases), occupation group (36 cases), preinjury mental health and substance use condition (13 cases), preinjury disability (124 cases). Significant associations are emphasised in bold.

Table S7. GOS-E independent function outcome descriptive statistics and estimated mean differences, N = 2537.

		GOS-E - Independent Recovery				
		**6-Months n (%)**	**12-Months n (%)**	**24-Months n (%)**	**OR (95%CI)**	**AOR (95%CI)**
MVA, latent class	1	193 (32.9)	224 (38.6)	201 (37.8)	1.00	1.00
	2	154 (30.1)	187 (36.7)	179 (37.5)	0.98 (0.68, 1.43)	1.06 (0.71, 1.58)
	3	158 (27.6)	185 (33.5)	173 (36.9)	0.77 (0.53, 1.11)	1.09 (0.71, 1.67)
	4	92 (29.2)	98 (32.0)	94 (36.9)	0.88 (0.57, 1.36)	1.09 (0.69, 1.73)
	5	28 (23.9)	25 (21.4)	26 (27.1)	**0.43 (0.23, 0.81)**	0.93 (0.45, 1.96)
Age (years)	15 to 24	149 (33.1)	180 (40.1)	183 (47.8)	1.00	1.00
	25 to 34	89 (25.0)	86 (25.4)	87 (29.2)	**0.27 (0.17, 0.43)**	**0.24 (0.15, 0.39)**
	35 to 44	78 (20.6)	97 (26.5)	84 (28.6)	**0.22 (0.14, 0.35)**	**0.21 (0.13, 0.34)**
	45 to 54	65 (20.7)	74 (24.1)	73 (26.6)	**0.19 (0.12, 0.31)**	**0.21 (0.12, 0.35)**
	55 to 64	75 (25.9)	87 (30.1)	93 (35.6)	**0.41 (0.25, 0.65)**	**0.32 (0.19, 0.55)**
	65 to 74	110 (43.7)	121 (48.8)	99 (42.3)	1.53 (0.93, 2.53)	0.66 (0.36, 1.21)
	75+	125 (41.4)	141 (47.3)	120 (42.3)	1.40 (0.89, 2.23)	0.55 (0.29, 1.02)
Sex	Male	425 (28.8)	476 (32.8)	463 (36.0)	1.00	1.00
	Female	266 (30.8)	310 (36.6)	276 (37.2)	1.22 (0.93, 1.59)	0.86 (0.64, 1.14)
Preferred Language, English	not English	15 (28.8)	15 (27.8)	14 (29.2)	1.00	1.00
	English	569 (30.9)	632 (35.2)	581 (37.0)	1.66 (0.66, 4.14)	1.53 (0.60, 3.89)
Residential Area	Regional and remote areas	253 (28.5)	288 (33.1)	276 (35.7)	1.00	1.00
	Major cities	432 (30.2)	490 (35.0)	456 (36.8)	1.11 (0.84, 1.45)	0.78 (0.57, 1.06)
IRSAD (quintile)	1, highest level of disadvantage	134 (28.1)	148 (31.8)	135 (32.0)	1.00	1.00
	2	123 (25.5)	152 (32.6)	139 (33.5)	1.03 (0.68, 1.56)	1.28 (0.84, 1.95)
	3	138 (28.5)	155 (31.7)	151 (35.3)	1.16 (0.77, 1.75)	1.23 (0.82, 1.85)
	4	131 (30.3)	154 (36.2)	147 (39.7)	**1.53 (1.01, 2.34)**	**1.70 (1.10, 2.62)**
	5, highest level of advantage	159 (35.9)	169 (39.8)	160 (42.6)	**2.24 (1.46, 3.43)**	**1.95 (1.24, 3.08)**
Education level	Did not complete high school	245 (30.5)	261 (33.5)	237 (34.0)	1.00	1.00
	Advanced diploma	183 (25.1)	217 (30.3)	219 (34.3)	0.78 (0.57, 1.07)	1.03 (0.74, 1.44)
	Completed high school	109 (32.0)	132 (39.2)	117 (42.4)	1.48 (0.98, 2.24)	1.50 (0.99, 2.29)
	University	131 (36.9)	143 (41.6)	135 (44.4)	**2.02 (1.35, 3.02)**	**2.31 (1.51, 3.54)**
CCI conditions	Yes	172 (25.8)	194 (30.1)	176 (30.0)	1.00	1.00
	No	519 (31.0)	592 (35.8)	563 (39.0)	**1.78 (1.34, 2.38)**	**1.97 (1.39, 2.78)**

		GOS-E - Independent Recovery				
		6-Months n (%)	12-Months n (%)	24-Months n (%)	OR (95%CI)	AOR (95%CI)
Pre-injury mental health condition	No	632 (30.2)	715 (34.8)	675 (37.1)	1.00	1.00
	Yes	50 (21.9)	62 (27.9)	58 (30.1)	**0.47 (0.30, 0.73)**	0.67 (0.41, 1.10)
Pre-injury substance use condition	No	628 (29.7)	719 (34.7)	662 (36.4)	1.00	1.00
	Yes	54 (25.6)	58 (28.2)	71 (37.0)	0.76 (0.50, 1.16)	1.32 (0.81, 2.15)
Pre-injury disability	No	581 (30.3)	624 (35.6)	574 (37.3)	1.00	1.00
	Yes	101 (26.0)	112 (31.4)	109 (34.9)	**0.69 (0.48, 0.98)**	**0.41 (0.27, 0.61)**
Occupation skill level/status	Elementary/labourers	44 (17.5)	56 (22.4)	52 (23.6)	1.00	1.00
	Intermediate	75 (22.7)	85 (25.9)	82 (29.7)	1.67 (0.97, 2.85)	**2.14 (1.21, 3.79)**
	Trade/ advanced clerical	88 (23.6)	113 (30.5)	115 (36.6)	**2.44 (1.44, 4.13)**	**1.98 (1.14, 3.44)**
	Professionals	113 (26.3)	137 (33.5)	122 (33.4)	**2.67 (1.61, 4.44)**	**2.57 (1.48, 4.45)**
	Not working	328 (41.3)	336 (43.1)	310 (43.1)	**7.77 (4.84, 12.48)**	**8.21 (4.72, 14.28)**
	Studying	34 (26.0)	51 (40.2)	53 (48.2)	**5.03 (2.50, 10.12)**	**2.37 (1.15, 4.89)**
Fault attribution	Another at fault	103 (24.3)	115 (28.0)	106 (29.4)	1.00	1.00
	Claim another at fault	27 (25.5)	32 (28.1)	34 (32.1)	1.06 (0.56, 2.03)	0.93 (0.47, 1.88)
	No other at fault	401 (31.2)	479 (38.3)	442 (40.4)	**2.51 (1.76, 3.59)**	**1.97 (1.25, 3.12)**
	Deny another at fault	16 (33.3)	15 (31.3)	14 (35.9)	1.56 (0.59, 4.11)	1.19 (0.41, 3.43)
	Unknown if another was at fault	144 (30.2)	145 (30.8)	143 (33.5)	**1.57 (1.04, 2.39)**	1.56 (0.94, 2.59)
ISS (tertiles)	1–10	271 (33.9)	295 (38.0)	230 (37.5)	1.00	1.00
	11–17	284 (32.8)	308 (36.2)	301 (38.6)	0.93 (0.68, 1.27)	**0.46 (0.31, 0.69)**
	18–75	136 (20.1)	183 (27.4)	208 (32.8)	**0.38 (0.27, 0.53)**	**0.15 (0.09, 0.25)**
Injured body regions	Orthopaedic injuries	182 (30.4)	192 (32.9)	173 (35.9)	1.00	1.00
	Chest/abdominal (with/without additional injuries)	273 (30.0)	323 (36.0)	322 (38.4)	1.19 (0.86, 1.66)	**3.30 (2.06, 5.29)**
	head injury	76 (29.0)	83 (31.8)	94 (38.4)	1.03 (0.66, 1.63)	**4.48 (2.45, 8.17)**
	SCI	<5	<5	<5	**0.01 (0.00, 0.48)**	**0.05 (0.00, 0.75)**
	other/multi	159 (29.3)	186 (35.3)	149 (34.1)	1.04 (0.72, 1.52)	1.37 (0.92, 2.03)
Months post-injury	6 months	691 (29.5)			1.00	1.00
	12 months		786 (34.2)		**1.57 (1.31, 1.89)**	**1.57 (1.31, 1.89)**
	24 months			739 (36.4)	**1.97 (1.60, 2.41)**	**1.97 (1.60, 2.41)**

Notes: Missing data were imputed for the following covariates: latent class (253 cases), preferred language (497 cases), residential area and IRSAD (28 cases), education level (134 cases), occupation group (38 cases), preinjury mental health and substance use condition (21 cases), preinjury disability (230 cases). Significant associations are emphasised in bold.
